# Determinants of Medical Practice Variation Among Primary Care Physicians: Protocol for a Three Phase Study

**DOI:** 10.2196/18673

**Published:** 2020-10-20

**Authors:** Sagi Shashar, Shlomi Codish, Moriah Ellen, Ehud Davidson, Victor Novack

**Affiliations:** 1 Clinical Research Center Soroka University Medical Center and Faculty of Health Sciences Ben-Gurion University of the Negev Beer-Sheva Israel; 2 Soroka University Medical Center and Faculty of Health Sciences Ben-Gurion University of the Negev Beer-Sheva Israel; 3 Department of Health Services Management Guilford Glazer Faculty of Business and Management Ben Gurion University Beer-Sheva Israel; 4 Institute of Health Policy Management and Evaluation Dalla Lana School of Public Health University of Toronto Toronto, ON Canada; 5 McMaster Health Forum McMaster University Hamilton, ON Canada; 6 General Management Clalit Health Services Tel Aviv Israel

**Keywords:** medical practice variation, variation determinants, primary care physicians, personal behavioral characteristics

## Abstract

**Background:**

One of the greatest challenges of modern health systems is the choice and use of resources needed to diagnose and treat patients. Medical practice variation (MPV) is a broad term which entails the differences between health care providers inclusive of both the overuse and underuse. In this paper, we describe a 3-phase research protocol examining MPV in primary care.

**Objective:**

We aim to identify the potential targets for behavioral modification interventions to reduce the variation in practice patterns and thus improve health care, decrease costs, and prevent disparities in care.

**Methods:**

The first phase will delineate the variation in primary care practice over a wide range of services and long follow-up period (2003-2017), the second will examine the 3 determinants of variation (ie, patient, physician, and clinic characteristics), and attempt to derive the unexplained variance. In the third phase, we will assess a novel component that might contribute to the previously unexplained variance - the physicians’ personal behavioral characteristics (such as risk aversion, fear of malpractice, stress from uncertainty, empathy, and burnout).

**Results:**

This work was supported by the research grant from Israel National Institute for Health Policy Research (Grant No. 2014/134). Soroka University Medical Center Institutional Ethics Committee has approved the updated version of the study protocol (SOR-14-0063) in February 2019. All relevant data for phases 1 and 2, including patient, physician, and clinic, were collected from the Clalit Health Services data set in 2019 and are currently being analyzed. The evaluation of the individual physician characteristics (eg, risk aversion) by the face-to-face questionnaires was started on 2018 and remains in progress. We intend to publish the results during 2020-2021.

**Conclusions:**

Based on the results of our study, we aim to propose a list of potential targets for focused behavioral intervention. Identifying new targets for such an intervention can potentially lead to a decrease in the unwarranted variation in the medical practice. We suggest that such an intervention will result in optimization of the health system, improvement of health outcomes, reduction of disparities in care and savings in cost.

**International Registered Report Identifier (IRRID):**

DERR1-10.2196/18673

## Introduction

### Background

Health care spending worldwide continues to increase and now accounts for approximately 17% of the gross domestic product in the United States, 9.8% in the Organisation for Economic Co-operation and Development (OECD) countries, and 7.5% in Israel [1]. Most experts consider the level of health care spending in the United States unsustainable [2]. Health economists identify unnecessary diagnostic and screening tests as a primary driver of this spending [2-5]. Moreover, many studies have shown that overuse neither benefits health care nor health outcomes [6-9] and may have adverse effects [10], leading to more unnecessary tests and treatment [11]. Major attempts to prevent the overuse of health services (HSs) include the British Medical Journal series *Too Much Medicine* [12], established in 2002, and *Choosing Wisely* campaigns [13], established in 2012. Increasing interest in this area is also reflected by the growing number of books, literature, and articles in the mainstream media [14].

### Medical Practice Variation

**Figure 1:**
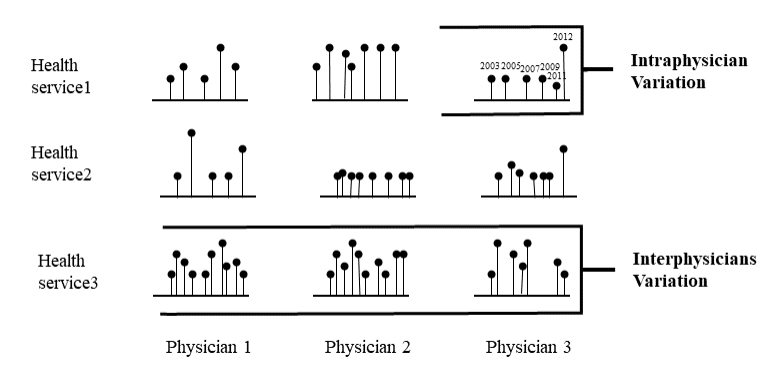
Two types of variation.

In this study we chose to investigate medical practice variation (MPV), coined by the Dartmouth research group [15,16]. While MPV is based on a relative comparison between providers (Figure 1), overuse and underuse definitions call for a comparison between the individual provider practice and standard of care (absolute comparison) based on a “gold standard” or guideline recommendations [16,17]. Both overuse and underuse (eg, patients not receiving optimal care and resources used inefficiently) have negative consequences [14] and can contribute to MPV [15]. MPV is associated with poorer health outcomes, increased costs, disparities in care, and burden on medical systems [14,17-21]. Adopting the policy aimed to reduce variation is a central theme of quality management that has begun with industrial production and was recently adopted in medicine practice [19,22,23].

Not all MPV has pernicious effects. One should distinguish between unwarranted and warranted MPV. Warranted MPV reflects patient-centered care as it takes into account patient differences such as clinical or patient-preference differences [20,24]. Unwarranted MPV is caused by many factors such as variable access to resources or differing physician opinions and preferences [15,16]. In our study, we try to focus on factors contributing to the unwarranted MPV.

### MPV Determinants

MPV causes can be divided into 3 main domains: patient characteristics, health care system characteristics, and physician characteristics [25,26]. Existing research suggests that variation is mainly attributable to patient characteristics, rather than to physician, or clinic [27-31]. Patient-related factors frequently studied in primary care [32] included age [33,34], type and severity of illness [33-35], socioeconomic status [36-39], ethnicity/race [40-42], and expectations of treatment effect [34,35,43,44]. Clinic factors included size [38,39,45,46], workload [46-48], funding method [49], services availability [35,50,51], services cost [52,53], and rural/urban location [15,34,54-56].

Factors related to the primary care physicians can be divided into demographic/professional and psychological characteristics. Demographic/professional characteristics include age, gender [33,42,46,47,57,58], specialty [38,58], area of expertise [36,47,57,59], and years of practice [33,46,58,60,61]. The psychological characteristics are discussed further on in the manuscript.

### Unexplained Variance

It appears that the major part of the variation is unexplained [31,62,63], that is, there are more influential factors that were not adequately estimated and researched so far [64], such as system-level and physician-level psychological characteristics. Assessing the extent of the variance that can be explained by the physicians’ psychological characteristics is essential because it will allow us to develop behavior modification tools that might help in reducing MPV [25]. Targeted interventions that address these variables might successfully optimize test ordering. The physician psychological characteristics include personality [65], attitudes [66], or behavior [67].

There is no generally agreed upon definition of personality, yet it is defined as person’s stable feelings, thoughts, and behavioral patterns [68]. The Big Five dimensions of personality are openness, conscientiousness, extraversion, agreeableness, and neuroticism [69]. Attitude refers to one’s opinions, beliefs, and feelings about aspects of his/her work environment. Two job attitudes have the greatest potential to influence how people behave at work: job satisfaction (feelings people have toward their job) and organizational commitment (the emotional attachment people have toward the company they work for) [68].

Compared with personality and attitudes, behavior is less abstract and more measurable, objective, and quantified and also it encompasses the other variables as it is a derivative of them [67,70]. Therefore we chose to focus on the physicians’ behavior characteristics. Four types of behaviors have been extensively studied in the organizational behavior literature: job performance, organizational citizenship behaviors, absenteeism, and turnover [68]. Our research, which studies the referral rates of the physicians, is related to the job performance behavior. Job performance refers to the success that one accomplishes in the tasks listed in his/her job description. Factors related to a physician’s job performance and medical decision making are the way s/he is treated at work, cognitive shortcuts (heuristics), the level of stress experienced at work, work attitudes to risks, and emotion [68,70].

### Personal Behavioral Characteristics

Personal behavioral characteristics studied to date with respect to unwarranted variation included risk attitudes [58,71-74], adherence to treatment guidelines [50,75-78], empathy [79], and fear of malpractice [80]. We believe that the comprehensive approach where we will investigate the effect of risk aversion, stress due to uncertainty, fear of malpractice, empathy, and burnout will bring a higher level of inference as each can be reliably measured and may uniquely and independently account for significant MPV.

#### Risk Aversion

Risk aversion is the tendency to minimize risk by choosing known options with more certain, but less beneficial, expected outcomes [81]. A risk averse physician, for example, might refer a patient for tests with an unclear, yet not urgent clinical presentation, despite the potential increase in cost and detrimental effect of unnecessary test. MPV studies show that longer cardiopulmonary resuscitation [74], higher use of laboratory services [71,72,82,83], more referrals [58,74], and higher admission rate to an intensive care unit [84] are associated with higher risk aversion. None to date, however, have examined the proportion of variance in the decision making that can be explained by risk aversion level.

We believe that the increased understanding of the mechanisms of risk-taking and risk-aversion behavior (eg, reward sensitivity, impulsiveness, and social anxiety) may suggest ways in which intervention programs can be designed and administered to be sensitive to individual differences between the physicians [85].

#### Stress From Uncertainty

Uncertainty is common among physicians who must make decisions based on incomplete and imperfect data, with unpredictable patient responses to testing and treatment [86]. Primary care physicians experience more uncertainty than specialists due to the breadth and complexity of scope, generalist orientation, focus on continuity, and psychosocial factors [87]. Previous research has shown that physician uncertainty is associated with MPV [32,87,88], yet again the extent to which it explains variance in the practice patterns has not been studied.

#### Fear of Malpractice Claims

Defensive medicine is defined as the ordering of tests, procedures, and patient visits for the purpose of averting malpractice [89]. A nationwide study of Israeli physicians concluded that defensive medicine is prevalent, mostly resulting in unnecessary tests, referrals to consultants, and hospitalizations [90]. Primary care physicians have historically experienced low rates of malpractice claims, attributed to the high regard for them in their communities, low numbers of invasive procedures, and mutual trust and communication developing with patients over time. In the most recent Medscape Malpractice report [91], primary care was not on the list of the top 10 specialties for lawsuits. However, recent years have seen an increase in the incidence of the malpractice claims in primary care [92]. Therefore, estimating the contribution of malpractice to MPV may be important as it may influence the physicians’ practice patterns and thus the variation between them.

#### Burnout

Burnout is increasing among general physicians [93] and associated with self-reported errors among primary care physicians and longer consultations [94]. However, it has neither been associated with overuse nor examined as a determinant of MPV. We assume that physicians with higher levels of burnout may have less variation in their HS utilizations and may be overused. This is because their discretion is consistently influenced by their mood and lack of motivation than by their patients’ medical needs.

#### Empathy

It has been shown that perceptions of patient needs, feelings, and primary care physicians’ ability to recognize emotions affect how they order tests [95]. For instance, physicians rated higher for empathy had a greater preference for intubation, ordered more laboratory tests, and performed cardiopulmonary resuscitation for longer periods [79]. Yet, it has not been proved as a determinant of MPV.

### Why Primary Care?

While most MPV research has focused on secondary and tertiary care in health regions and hospitals [15], this study examines MPV across primary care physicians. Primary care accounts for 14% of all health care spending on average across OECD countries with patient–physician consultations accounting for the majority (55% in the United States; 90% in the UK) [96]. Determining what accounts for MPV in primary care can help to develop targeted approaches for preventing unnecessary tests and treatment, better care coordination, cost containment, and improved health outcomes.

### Israel’s Health System

In 2019, Israel was ranked the 10th healthiest country in the world by Bloomberg rankings, out of 169 countries [97]. The National Health Insurance Law of 1995 mandates all citizens resident in the country to join 1 of the 4 official not-for-profit health maintenance organizations, which are prohibited by law from denying any Israeli resident a membership [98]. The study is placed in the Southern District of Israel, the Negev, and includes physicians and patients of the Clalit Health Services health maintenance organizations, the largest health insuring organization in Israel (4.5 million insurees). Clalit Health Services is the largest health care provider in the area, covering approximately 70% of a population of 730,000 residents in the Negev.

### Health Services

In this study we aimed to analyze HSs in the primary care in situations where the physician has the freedom of action to decide whether to utilize them [99], that is, clinical scenarios with discretionary decisions [100]. For instance, referring a patient with ST-elevation myocardial infarction to the hospital is not a discretionary decision as the physician’s findings reflect an undoubtable urgent condition of myocardial infarction which can be cured in a catheterization room. But ordering a thyroid-stimulating hormone (TSH) test for a patient with generalized weakness is discretionary, because weakness is a nonspecific symptom which can be caused by many factors such as an infection, anemia, inflammation. For these discretionary HSs, different choices carry different benefits and risks and therefore we believe that physicians will differ in the decisions [101,102], based on their knowledge, experience, beliefs, and thoughts. We hypothesize that the derivative of these components, the physician personal behavioral patterns, can significantly influence the utilization of the services in this category. Therefore, we identified the 16 most frequently utilized HSs in primary care that can be discretionary.

- Four imaging tests: bone scans, computed tomography of the brain and spine, chest x-ray, magnetic resonance imaging;
- A composite of cardiac tests including Holter electrocardiography (ECG), echocardiogram, stress test, and transesophageal echocardiography;
- Six laboratory tests: vitamin B_12_, vitamin D, TSH, hemoglobin, carcinoembryonic antigen, prostate-specific antigen;
- Three specialist consultation visits: rheumatology, pulmonary, and neurology;
- Two emergency department visits due to chest pain or back pain.

### Objectives and Hypothesis

**Figure 2:**
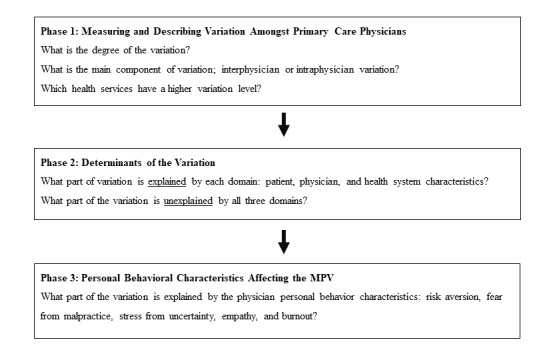
Study flowchart - medical practice variation assessment.

This paper describes a 3-phase research protocol (Figure 2) of MPV of primary care physicians across 16 HSs in the largest health care network in Southern Israel (Clalit Health Services) between 2003 and 2017.

The study’s objectives are to (1) describe the variation of HSs referrals among primary care physicians; (2) derive the unexplained variance after the adjustment for patient, physician, and clinic characteristics; (3) assess the extent to which the personal behavioral characteristics of the primary care physicians contribute to the explanation of the unexplained variance; and (4) identify the potential targets for behavioral modification interventions to reduce the variation in practice patterns and thus improve health care, decrease costs, and prevent disparities in care.

We hypothesize that the physicians’ personal behavioral characteristics such as risk aversion, stress due to uncertainty, fear of malpractice level of empathy, and burnout are affecting the decision-making process as demonstrated by the different levels of their HSs referrals.

### Patient and Public Involvement

This research was done without patient involvement. Patients were not invited to comment on the study design and were not consulted to develop patient-relevant outcomes or interpret the results. Patients were not invited to contribute to the writing or editing of this document for readability or accuracy.

## Methods

### Study Phases

The first phase will delineate the variation in primary care practice over a wide range of services and long follow-up period, the second will examine the 3 determinants of variation (ie, patient, physician, and clinic characteristics) and attempt to derive the unexplained variance. In the third phase, we will assess a novel component that might contribute to the previously unexplained variance—the physicians’ personal behavioral characteristics such as risk aversion, fear of malpractice, stress from uncertainty, empathy, and burnout.

### Data Collection

Data will be collected from the computerized medical records of Clalit Health Services and will include (1) patient data (age, sex, marital status, residence type [urban, rural, or nomadic], number of annual visits by community physicians, background diseases, and socioeconomic status according to address); (2) primary care physician data (age, sex, years of active practice, area of expertise, specialty, country of birth, familial status, ethnicity, country where medical studies were completed, and number of insured patients in the physician’s unit); (3) clinic data (number of insured patients per doctor, number of physicians per 1000 patients, number of annual visits, location [rural/urban]); (4) HS data (annual referral number per physician per clinic for bone scan, computed tomography, chest x-ray, magnetic resonance imaging, Holter ECG, echocardiogram, stress test, transesophageal echocardiography, vitamin B_12_ test, vitamin D test, TSH test, hemoglobin test, carcinoembryonic antigen test, prostate-specific antigen test; specialist consultation visits for rheumatology, pulmonary, and neurology; and emergency department visits due to chest pain and back pain).

The unit of analysis is physician/clinic/year. Each patient is assigned to 1 primary care physician and thus all data will be assigned annually to a physician per clinic. The HSs data will be extracted by numerical ID codes given for each HS.

### Phase 1: Measuring and Describing Variation Among the Primary Care Across 16 HSs

In this initial phase of the research, we set out to identify MPV patterns by describing and comparing variation between HSs. Our first objective is to identify the main component of variation, that is, which variation is larger, between physicians or within the physician over time (interphysician vs. intraphysician variation). Additionally, we sought to identify which HSs have the highest variation and investigate their common characteristics.

The interphysician variation is the difference in utilization rates between physicians for a given HS, and the intraphysician or within-physician variation is the difference in practice pattern of an individual physician over the years. The difference between the 2 is illustrated in Figure 1. Most research to date examines variation in practice behavior between health care providers at one point in time, not within-physician variation over time. By delineating the 2 (between vs within), we can determine which accounts for a greater proportion of MPV (ie, relative importance of between- and within-physician variation) and what factors predict each. This information is germane to the policy makers, helping them determine where to direct efforts to reduce MPV. For instance, if within-physician variation accounts for a significant proportion of MPV, resources and efforts should be directed at fostering consistent physician clinical behavior over time. However, if between-physician variation is greater, efforts should be directed to assist overusing and underusing physicians to accommodate their utilization patterns to the appropriate level.

#### Statistical Analysis: Coefficient of Variation

Operational definitions and methods used to describe variation are diverse and inconsistent. Different authors have defined variation as absolute values [103,104]; rates [105,106]; ratios between tests [107,108] or 90th/10th percentiles [108,109]; and percentages of the overuse/underuse [96,110] or utilization [109,111] or inappropriate use [112]. We instead will measure variation using the coefficient of variation (COV), which is the ratio of the SD to the mean (SD/mean × 100). It represents the percent of the dispersion out of the central tendency, where higher values indicate larger difference between values (ie, higher variation). The COV is a standardized measurement; however, determining high variation for low-utilized HSs is considered overestimation [26], and therefore should be interpreted with caution.

The numerical levels (threshold) of COVs defining high versus low utilization differ across fields of science [113,114] and are not defined in MPV research literature [115]. Because it is a frequently used measure in the field of health policy research, there is a need for a consensus as to what represents high or low variation for each particular service [116].

To compare the utilization levels between physicians in each HS, we plan to calculate annual utilization rates per 1000 patients: ([utilization levels/total insured patients affiliated to the physician] × 1000 patients). Further, for each HS we will calculate between-physician COVs based on the averaged physicians’ rates and within-physicians’ COVs by the averaged individual physicians rates over the years of practice.

#### Correlation Between Variation and Utilization Levels

To identify potential MPV patterns, we will examine correlations between the averaged between- and within-physician variations and utilizations at the HSs level. For this analysis, we will use the Spearman test and chart the HSs’ averaged utilizations and COVs to enable visual comparisons.

In conclusion for this phase, we intend to describe the MPV pattern among primary care physicians, identify the source of a greater variation component (between vs within), and the HSs with higher variation and higher utilization rates.

### Phase 2: Determinants of the Variation

After describing the MPV patterns in primary care, we will then assess its determinants. The primary objective of the second phase is to estimate the extent to which each determinant explains the variation and to deduce the overall unexplained variance (Figure 2). In this stage we will collect a wide range of variables related to the domains, calculate the adjusted variance, and the proportional change in the variance (PCV) [117].

#### Statistical Analysis

PCV will be computed to determine the proportion of variance accounted for by each domain (patient, physician, clinic) across all HSs, using the following formula: PCV=(Vn1–Vn2)/Vn1. First, for each HS we will calculate the crude variance (Vn1), then, we will compute 3 regression models, each including covariates related to the domain. According to the models’ predicted values, we will calculate the adjusted variance (Vn2), expecting a decrease from the crude variance. Then, for each HS we will calculate 3 PCVs, 1 for each domain, assessing the percent of the variance explained by each domain. The larger the PCV (ie, the larger the difference between crude and adjusted variances), the greater the variance explained by that domain. Consequently, we will determine which domain explains most of the variance across all HSs, and will be able to estimate the overall unexplained variance.

#### Prediction Model

As previously mentioned, we will perform regression model analysis to derive the adjusted variances for patient, physician, and clinic characteristics. We will utilize generalized linear negative binomial mixed models, the annual HSs utilizations as outcomes (nominator), and the annual insured patients per physician as the outcome’s offset (denominator), and thus, defining the rates, the “count” variable of the negative binomial distribution. Physicians, clinics, and years (to account for secular and trajectory trends) will be defined as random effect clusters and patient, physician, and clinic characteristics will be included as fixed covariates separately. We will use “glmmTMB” R package (R Foundation for Statistical Computing), version 1.0.136 and IBM SPSS, version 24 for statistical analysis.

### Phase 3: Personal Behavioral Characteristics Affecting the Variation

In this phase we will visit the clinics and ask the physicians to fill 5 short, validated research questionnaires measuring risk aversion, stress from uncertainty, fear from malpractice, empathy, and burnout [74,118]. We assume that these behavior characteristics are substantially stable [119,120] as well as the practice habits [121]. For instance, burnout [122] or stress from uncertainty is not a temporary emotion, but rather stable, as both are incurred by the properties of the specialty and the physician’s capabilities and characteristics which tend to be fixed [122]. However, to be aligned with the most accurate and updated behaviors and practice habits, we chose in phase 3 to include physicians who worked also during 2017 (approximately 180) as we started to interview them in 2018. Furthermore, we will exclude physicians who worked starting from 2017, as the within-physician variation cannot be assessed for them. To increase the response rate, we plan to conduct face-to-face sessions during which the physicians will be asked to complete the questionnaires. We expect to achieve a response rate of more than 75%.

#### Questionnaires Scales

The risk-taking scale is a validated subset of the Jackson Personality Index that measures general risk-taking behavior in emergency physicians [89,123,124] and has 6 items, each rated on a 6-point Likert scale. Possible scores range from 6 to 36, and higher scores correspond to increased risk-taking [125,126]. The stress due to uncertainty is a validated psychometric tool, with a Cronbach alpha of .90 [127], that measures physician’s stress due to uncertainty in patient care. It has 13 items, each rated on a 6-point Likert scale. Possible scores range from 13 to 78, with higher scores corresponding to higher stress due to uncertainty. The Malpractice Fear Scale is a validated scale, with a Cronbach alpha of .88 [93] that measures fear of malpractice in primary care and emergency physicians [58,123,128,129]. It has 6 items, each rated on a 5-point Likert scale, while possible scores range from 5 to 30, with higher scores corresponding to increased fear of malpractice. Empathy will be assessed by the Jefferson Scale of Physician Empathy, which consists of 20 items, with each rated on a 7-point Likert scale. Higher sum-scores indicate higher levels of empathy. The scale has been validated by explorative factor analysis and test–retest reliability [130]. Burnout will be assessed by the Maslach Burnout Inventory Human-Services-Survey [131], which has been used in more than 90% of empirical studies on burnout globally [132]. The Maslach Burnout Inventory – Human Services Survey consists of 22 items scored on a 7-point Likert scale constituting 3 subscales: (1) emotional exhaustion (9 items); (2) depersonalization (5 items); and (3) personal accomplishment (8 items). Burnout is defined as either a high score on the emotional exhaustion subscale or a high score on the depersonalization subscale or a low score on the personal accomplishment scale [131].

#### Statistical Analysis

During the analytic phase we will first compare the patient case mix and clinic and demographic characteristics between respondents and nonrespondents. This will allow us to estimate the degree of the potential bias in the analysis of the practice patterns. Then, we will assess the extent to which personal behavioral characteristics (ie, risk aversion, stress due to uncertainty, fear of malpractice, empathy, and burnout) contribute to the explanation of the unexplained variance defined in the previous phase, using the PCV approach. We hypothesize that high rates of HSs referrals will be associated with high levels of risk aversion, stress due to uncertainty, fear of malpractice, empathy, and burnout.

### Ethics Approval and Consent to Participate

The study was approved by the Soroka University Medical Center Institutional Ethics Committee (0063-14-SOR). The consent to participate was written as part of the questionnaires.

### Availability of Data and Material

The data sets that will be used or analyzed during this study will be available following local Ethics Committee approval.

## Results

This work is supported by the research grant from Israel National Institute for Health Policy Research (2014/134). The funding agency has no input on the study design or execution. Our study protocol has undergone peer review by the funding body.

Soroka University Medical Center Institutional Ethics Committee has approved the updated version of the study protocol (SOR-14-0063) named “Determinants of Medical Practice Variation among Primary Care Physicians,” in February 2019. The approval is valid until March 2021 and can be extended by request.

All the data for phase 1 (assessment of the cured variation) and phase 2 (derivation of the adjusted variation) including patient, physician, and clinic data were collected from the Clalit Health Services data set in 2019 and are currently being analyzed. The evaluation of the physicians’ personal behavioral characteristics by the face-to-face questionnaires (phase 3) was started in 2018 and remains in progress. We intend to publish the results during 2020-2021.

## Discussion

### Overview

This study will allow us to approach the challenge of the targeted MPV reduction policies by answering a number of questions: What is the degree of the variation and what services have higher variation rates? Which variation is larger: between the physicians or within the physicians over time? What part of the variation cannot be explained by the patient case mix, clinic characteristics, or professional characteristics of the physician? Can physician personal behavioral characteristics explain part of the variation?

### Risks and Limitations

Our current study focuses on the variation in the practice patterns, yet we cannot infer the clinical appropriateness of the HSs used. In more general terms we will be not able to deduce what physician is practicing “good” medicine—the one who sends patients to a lot of tests or the one who sends few. Yet, because MPVs have been previously shown to be associated with poorer health outcomes [14,17-19,21], we believe that focusing on the measurement and dissection of the variation itself can contribute to the development of the approaches to reduce the MPV.

Another major limitation of the variation research in medicine is that acceptable values for variation are not defined. Therefore, we will be able only to have a relative comparison identifying factors associated with a higher variation (eg, physician characteristics, specific services).

Furthermore, in some circumstances the utilization of the HSs we are assessing can be considered not discretionary; for example, emergency department visits due to ECG presenting ST elevations in the primary care clinic, vitamin B_12_ laboratory tests for macrocytic anemia, or chest X-rays for chest injuries. However, the inclusion of these events decreases the variation and thus results in the bias toward zero (null hypothesis).

Moreover, our study precludes system-level factors such as resource constraints, process, workflow issues, funding, services accessibility. This is because we chose to focus on the physician level, not the system level. Therefore, we are only including patients and physicians belonging to 1 health care network (Clalit Health Services), thereby controlling for some of the system-level variation. However, it is possible that the variation between health care networks in the public health system in Israel is limited as the law controls their services’ provision and accessibility, and thus reduces disparities in care. These health care networks are not-for-profit and are prohibited by law from denying any Israeli resident a membership. Yet, between regions in Israel there is a variation in the mentioned factors and therefore our findings can be generalized onto other regions and countries only after accounting for the patterns of this region.

At the final stage of our research we aim to identify behavioral characteristics associated with a higher variation by applying validated questionnaires. Physicians’ attitudes as assessed by the abstract questionnaires may not fully represent their action in real-life clinical practice. Future research should aim at developing more reliable tools for assessing behavioral components of physicians practice.

The conventional risk of questionnaire-based research is a low response rate. To address this concern, we schedule face-to-face sessions with each one of the physicians enrolled into the study.

### Outcomes of the Research Program

We expect to analyze a total of 3 million patient years and 6.5 million test utilizations across 16 diverse HSs, referred by 250 physicians in 170 clinics, over 15 years of practice. The size and comprehensiveness of the data will provide a good reassurance for the robustness and generalizability of the research program.

Focusing on the physician personal behavioral characteristics as a major contributing factor to the variation is essential, because it may allow us to identify what are the likely sources of unwarranted variation that can be redressed. We believe that most of the variation explained by patient or clinic characteristics is generally reasonable (eg, greater use by ill and older patients) [20]. However, variation stemming from the physician personal behavior characteristics might be unwarranted and can be reduced without negatively affecting patient care.

Based on the results of our study, we aim to propose a list of potential targets for focused behavioral intervention. The research of behavioral interventions designed for physicians is limited, and describes only a handful of strategies. The most common approaches focusing on changing the practice habits include clinical decision support, shared decision making, pay-for-performance, and insurer restrictions [133]. We believe that identifying new targets for such an intervention during the digital health era can potentially lead to a decrease in the unwarranted variation in the medical practice and thus to the improvement of health outcomes, reduction of disparities in care, and cost savings.

## Appendix

Multimedia Appendix 1 Peer-review reports.

## Abbreviations

CHS: Clalit Health services
HS: health service
MPV: medical practice variation
TSH: thyroid-stimulating hormone
