# Supplementary material for: Determinants of Medical Practice Variation Among Primary Care Physicians: Protocol for a Three Phase Study
Source: JMIR Res Protoc. 2020 Oct 20;9(10):e18673. doi: 10.2196/18673 (PMC7609196; doi:10.2196/18673)
Supplement: Multimedia Appendix 1 [file resprot_v9i10e18673_app1.pdf]

הצעת המחקר קשורה לתחום של "בחירה נבונה" ובמסגרתה מעוניינים החוקרים לבחון את השימוש בבדיקות אבחנתיות. בשלב ראשון החוקרים מציעים לבחון את ההפניות של כלל רופאי הקהילה עבור כלל המבוטחים של קופת חולים כללית באזור הדרום בגיל 18 ומעלה במשך עשור בחמישה תחומים: בדיקות דימות, בדיקות מעבדה, הפניה לרפואה דחופה, הפניה לרופאים מומחים והפניה לבדיקות אבחון לבביות. בשלב שני של המחקר מצעים החוקרים לבחור עבור כל תחום את "שלושת השירותים השכיחים ביותר שעבורם ניתן לשייך מדד תוצא בריאותי".

כך, בשלב זה ההצעה היא כללית למדי ואינה מפרטת מהן הבדיקות או ההפניות בהם יעסקו החוקרים. היה רצוי מלכתחילה, על סמך מידע קיים, ספרות וכד'- להציע אוסף ממוקד יותר של בדיקות או הפניות, לפרט את הרציונל לבחירת הבדיקות או הפרוצדורות (כך למשל, במסגרת הקמפיין של בחירה נבונה מדובר בבדיקות המבוצעות בשכיחות גבוהה, יקרות יחסית, אשר עבורן הראיות בספרות הקיימת אינן מצביעות שיש בהן יתרון משמעותי לפחות לקבוצה משמעותית של חולים). אומנם השלב הראשון שהציעו החוקרים אמור לתת מידע על נושא השכיחות, אולם בעזרת מידע קיים וקריטריונים הולמים יכולים החוקרים לצמצם את אוסף התחומים, ההפניות והבדיקות בהם יעסקו, לפרט את סוגי החולים, משתני התוצאה וכו'.

בשלב הראשון של ניתוח הנתונים החוקרים מעוניינים לאתר גורמים ברמת הרופא, המרפאה והחולה הקשורים בבדיקות/הפניות. החוקרים מציעים לבצע ניתוח רב רמות ברמת הרופא תוך לקיחה בחשבון של רמת המרפאה. כדאי לשקול ניתוח רב רמות ברמת החולה, תוך כדי לקיחה בחשבון של רמת הרופא והמרפאה (שלוש רמות). לצרכי תיקנון האם יהיה שימוש באבחנות?

בשלב השני של ניתוח הנתונים החוקרים מעוניינים לקשר בין בדיקות/הפניות לבין תוצאי בריאות – האם תילקח בחשבון בדיקה אחת? האחרונה? הראשונה? מכלול הבדיקות ? לצרכי תקנון האם יהיה שימוש באבחנות/ רשמים ?

בהשערות המחקר כותבים החוקרים כי "קיימת שונות רבה בקשר שבין שיעור השימוש בשירותים לבין התועלת הקלינית שמפיקים החולים כתוצאה מכך". שונות בין מי למי? בין הרופאים המפנים? בין החולים? בין בדיקות שונות?

134-14 Rev I

The research proposal is related to the field of "choosing wisely" of which the researchers wish to examine the use of diagnostic tests. In the first phase, the researchers propose to examine the referrals of all primary care physicians for all insurers at age 18 and over of Clalit HMO in the southern area for a decade of practice, in five areas: imaging tests, laboratory tests, emergency medicine referrals, referrals to specialist physicians and referrals for cardiac diagnostic tests. In the second phase of the study, the researchers will select for each field the "three most common services for which a health outcome measure can be associated”.

Thus, at this stage, the proposal is fairly general and does not specify what tests or referrals the researchers will assess. In the first place, based on existing information, literature, etc., it would be desirable to propose a more focused collection of tests or referrals, to elaborate on the rationale for selecting the tests or procedures (for example, in the “choosing wisely” campaign, these are high-frequency, relatively expensive tests for which the existing literature indicates that they do not have a significant benefit to a large group of patients). Although the first phase suggested by the researchers should provide information on the prevalence issue, with the help of existing information and appropriate criteria, the researchers can reduce the range of domains, referrals and tests they will deal with, detail the types of patients, outcome variables, etc.

In the first phase of data analysis, the researchers are intending to identify physician, clinic, and patient-level factors related to tests / referrals. The researchers suggest to perform multi-level analysis at the physician level, taking into account the clinic level. You should consider multi-level patient-level analysis, taking into account the physician and clinic levels (three levels). Will the diagnosis be used for adjustment?

In the second phase of the data analysis the researchers will to assess the association between tests / referrals and health outcomes - will one test be taken into account? The latest? The first? All the tests? Will the diagnosis / preferences be used for adjustment?

In the hypotheses section of the proposal, the researches mention the "there is a great variation in the association between the rate of service utilization and the clinical benefit that patients derive as a result." Variation between who? Among the referring physcians? Among the patients? Among different tests?

1. הצעת המחקר מנוסחת ברמה כללית ולא ברור לאן היא תגיע, כלומר, אילו תחומים ואילו בדיקות ייבחנו במחקר. לטעמי, בחירת התחומים לא נכון שתהיה חלק מהמחקר, אלא צריכה להתבצע קודם לכן, מאחר שניתן להפיק את נתוני השימוש בבדיקות יחסית בקלות ולהחליט על הבדיקות שאותם בוחנים מראש. כך יהיה אפשר למקד את המחקר בצורה טובה יותר. כפי שהגיעו החוקרים לבחון במעין פיילוט את נתוני ויטמין B12, אפשר להחליט **מראש** על עוד בדיקות שכיחות בכל אחד מהתחומים אותם רוצים החוקרים לבחון (הדמיה, מעבדה וכו').
2. בעוד השלב השני של המחקר (איפיון הגורמים הקשורים לשיעור ההפניות) נראה נכון מתודולוגית, מעשי ובעל תרומה חשובה, לעניות דעתי המתודולוגיה של השלב השלישי של המחקר (הקשר בין שיעור ההפניה למדדי התוצא) מוטעית ביסודה ולא תוביל למסקנות כלשהן.
   א. ראשית רוצים החוקרים למצוא האם יש קשר בין שיעור ההפניה לבדיקה לתוצאה פתולוגית שלה. זהו לא מדד תוצא, אלא יכול להוות פרמטר עקיף ולא מדויק למוצדקות הבדיקה (אם כי הבדיקה יכולה להיות מוצדקת גם כאשר תוצאתה שלילית).
   ב. שנית רוצים החוקרים להעריך את תועלת הבדיקה על פי תוצאים ספציפיים בתקופה של לאחר הבדיקה (למשל, אירוע איסכמי לאחר בדיקת ארגומטריה) – לדעתי, לא ניתן יהיה מעשית להראות קשר כלשהו, מה גם שחישוב גודל המדגם לא בוצע בהתאם לחלק זה.
3. המלצתי היא למקד את המחקר בשלב השני שלו לאחר שנבחרו הבדיקות בהן רוצים להתמקד ולחקור את המאפיינים הקשורים לשיעור ההפניות, תוך הורדת החלק העוסק בתוצאים הקשורים לבדיקות.
4. יש לעדכן את ניתוח העלויות של המחקר בהתאם

134-14 Rev II

1. The research proposal is drafted at a general level and it is unclear where it will go, that is, which areas and tests will be tested in the study. In my opinion, choosing the areas should not be part of the research, but should be done earlier, since the data on the utilization of tests can be derived relatively easily and accordingly to decide on the tests that will be assessed in advance. This will allow to better focus the research. As the researchers analyzed at a kind of pilot study the vitamin B12 data, it is possible to decide in advance on more common tests in each area that the researchers want to examine (imaging, laboratory, etc.).
2. While the second phase of the study (characterizing the factors associated to the referral rates) seems to be methodologically correct, practically and important, in my opinion the methodology of the third phase of the study (the relationship between referral rates and outcome) is fundamentally wrong and will not lead to any conclusion.
A. First, the researchers want to find out whether the referral rate for a test is related to a pathological outcome. This is not a proper outcome, but can be an indirect and inaccurate parameter for the justification of the test (although the test can be justified even when its result is negative).
B. Secondly, the researchers want to evaluate the utility of the test according to specific outcomes in the post-test period (e.g., ischemic event after the ergometry test) - in my opinion, it is practically impossible to show any association, also because the sample size was not calculated according to this phase of the study.
3. It is my recommendation to focus the research in its second phase after selecting the tests that you want to focus on and estimate the characteristics related to the referral rates, while precluding the section dealing with the outcomes associated with the tests.
4. The estimated costs of the study should be updated accordingly.

האם לא לבצע קודם רצוי פיילוט על מנת לבדוק את נגישות וטיב המידע. האם כל המידע הנדרש באמת זמין?

134-14 Rev III
Isn’t it better to first perform a pilot to check the accessibility and quality of the data. Is all the required data really available?

1. **אני חושב שהמחקר המוצע ישיב לשאלת המחקר הראשונה (לאפיין את דפוסי ההפניה לשירותי בריאות נבחרים על ידי רופאי הקהילה ואת הגורמים הקשורים בכך). עם זאת, ברור הוא כי לרופאי קהילה שונים דפוסי הפניה שונים, ואינני מצפה לממצאים מפתיעים.**

2. **תוצאות הבדיקה של השאלה השנייה (להעריך את התועלת הקלינית שבהפניות על ידי הערכת הקשר בין שיעור ההפניות לבין שיפור בתוצאי הבריאות המותאמים לסוג ההפניה) והשלישית (לזהות גורמים הקשורים בתועלת רבה יותר משימוש בשירותי רפואה וכאלה הקשורים לתועלת נמוכה) תהיינה קשות לפרוש: ניתן לצפות מתאם חיובי בין שיעור ההפניות לבין חומרת המחלה. נראה לי כי חומרת המחלה תגרום להטיה בהערכת התועלת הקלינית שבהפניות. המחברים עדיין לא החליטו מה יהיו מדדי התוצאה הספציפיים אשר יהוו את המשתנים התלויים לבחינת הקשר בין שיעור ההפניה של הרופא לבין מדדי תוצאה אלו. אישית אני מתקשה לחשוב על תוצא שלא יהיה מושפע ע"י חומרת המחלה**

134-14 Rev IV

1. I think the proposed study will answer the first research question (to characterize the referral patterns for selected health services referred by primary care physicians and their associated factors). However, it is clear that different primary care physicians have different referral patterns, and I do not expect surprising findings.

2. The results of the second question (assessing the clinical benefit of the referrals by assessing the association between the referral rates and the improvement in health outcomes related to the referral type) and the third (identifying factors associated with greater benefit of utilizing health services and those associated with low benefit) would be difficult to interpret: positive correlation between the referral rates and disease severity is expected. It seems to me that the severity of the illness will bring to a bias in the assessment of the referrals' clinical utility. The authors have not yet decided what the specific outcome measures, which will be the dependent variables for examining the association between the physician referral rate and these outcome measures. Personally, I find it hard to think of an outcome that will not be affected by the severity of the disease
